# Supplementary material for: The interactome of intact mitochondria by cross-linking mass spectrometry provides evidence for coexisting respiratory supercomplexes
Source: Mol Cell Proteomics. 2017 Dec 8;17(2):216–32. doi: 10.1074/mcp.RA117.000470 (PMC5795388; doi:10.1074/mcp.RA117.000470)
Supplement: Supplemental Data [file supp_RA117.000470_133922_0_supp_23978_fzffwf.zip › spectra_annotation/mito_DR_spectra_annotation/22-1-12-1-2-1.pdf]

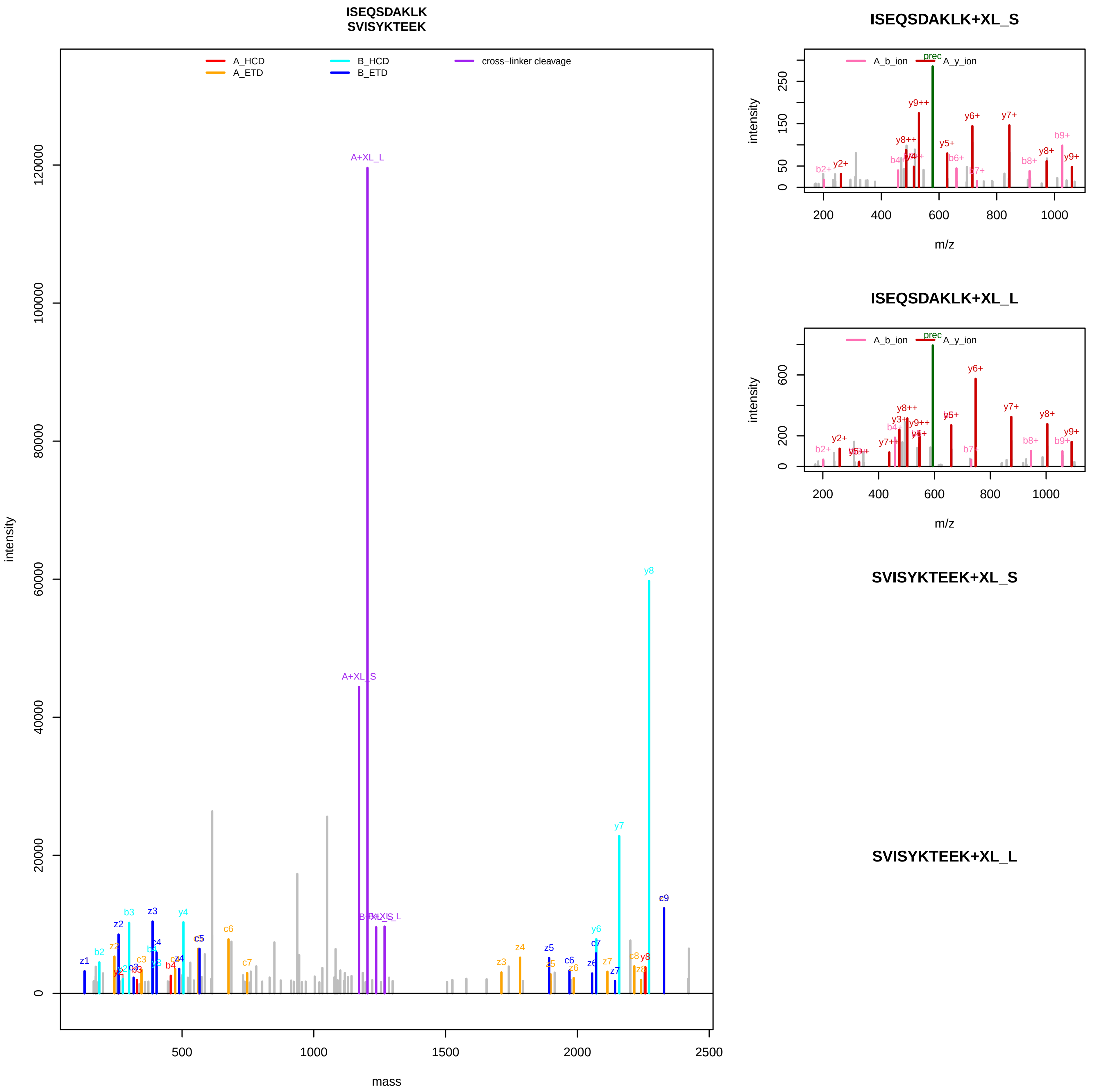

ISEQSDAKLK
SVISYKTEEK
A_HCD
A_ETD
B_HCD
B_ETD
cross−linker cleavage
A+XL_L
A+XL_S
y8
y7
c9
intensity
mass
ISEQSDAKLK+XL_S
A_b_ion
A_y_ion
prec
y9++
y6+
y7+
y5+
y8++
b9+
y8+
y9+
b8+
b6+
b7+
y2+
b2+
m/z
ISEQSDAKLK+XL_L
y6+
y8++
y7+
y8+
y9+
y3+
y9++
y5+
y4+
y7++
b4+
b8+
b9+
b7+
b2+
y2+
SVISYKTEEK+XL_S
SVISYKTEEK+XL_L
